# Corpus luteum number and maternal circulatory adaptation from early pregnancy onwards: the Rotterdam Periconception Cohort (Predict Study)

**DOI:** 10.1093/humrep/deaf181

**Published:** 2025-09-16

**Authors:** L W Voskamp, J J Koerts, R E Wiegel, K Verdonk, A H J Danser, R P M Steegers-Theunissen, M Rousian

**Affiliations:** Department of Obstetrics & Gynaecology, Erasmus University Medical Center, Rotterdam, The Netherlands; Department of Internal Medicine, Erasmus University Medical Center, Rotterdam, The Netherlands; Department of Obstetrics & Gynaecology, Erasmus University Medical Center, Rotterdam, The Netherlands; Department of Obstetrics & Gynaecology, Erasmus University Medical Center, Rotterdam, The Netherlands; Department of Obstetrics & Gynaecology, Erasmus University Medical Center, Rotterdam, The Netherlands; Department of Internal Medicine, Erasmus University Medical Center, Rotterdam, The Netherlands; Department of Internal Medicine, Erasmus University Medical Center, Rotterdam, The Netherlands; Department of Obstetrics & Gynaecology, Erasmus University Medical Center, Rotterdam, The Netherlands; Department of Obstetrics & Gynaecology, Erasmus University Medical Center, Rotterdam, The Netherlands

**Keywords:** corpus luteum, blood pressure, mean arterial pressure, uterine artery pulsatility index, uterine artery resistance index, circulation, assisted reproduction, IVF, ICSI outcome

## Abstract

**STUDY QUESTION:**

Is the number of corpora lutea (CL) associated with maternal circulatory adaptation to pregnancy, as assessed by blood pressure and uterine artery Doppler pulsatility and resistance indices?

**SUMMARY ANSWER:**

Pregnancies without a corpus luteum have a higher mean arterial pressure throughout pregnancy and lower uterine artery pulsatility and resistance indices in the first and second trimesters, compared to pregnancies where one or more than one corpus luteum is present.

**WHAT IS KNOWN ALREADY:**

Different modes of conception result in varying numbers of corpus luteum in early pregnancy. Previous research has demonstrated significant differences in hypertensive disorders of pregnancy and birthweight in women with 0, 1, and multiple CL, as well as altered maternal cardiovascular adaptation. Although direct causal evidence is limited, these differences are thought to reflect the presence or absence of corpus luteum-derived hormones, suboptimal decidualization in programmed cycles, or both.

**STUDY DESIGN, SIZE, DURATION:**

This prospective study used data from the ongoing Rotterdam Periconception Cohort, including women with singleton pregnancies enrolled from 2010 to 2022 at the Erasmus MC, University Medical Center, a tertiary care facility.

**PARTICIPANTS/MATERIALS, SETTING, METHODS:**

The study population for this research involved pregnancies in 1986 women: 1456 with one corpus luteum (1292 due to natural conception or insemination and 164 due to natural cycle frozen embryo transfer), 457 with more than one corpus luteum (due to fresh embryo transfer), and 73 with no corpus luteum (due to artificial cycle (AC)-FET). Linear mixed models were adjusted for maternal age, body mass index, nulliparity, smoking, pre-existing hypertension, and uterine artery Doppler outcomes, including mean arterial pressure.

**MAIN RESULTS AND THE ROLE OF CHANCE:**

Adjusted mean arterial pressure during pregnancy was significantly higher in women with 0 vs 1 CL (β + 2.19 mmHg, 95% CI [0.43–3.95], *P* = 0.015), but was not different between those with >1 and 1 CL (β −0.35 mmHg [−1.22 to 0.53], *P* = 0.438). This was also true for diastolic but not for systolic blood pressure. Uterine artery Doppler indices were available for 624 women. Adjusted uterine artery pulsatility index (PI) and resistance index (RI) were significantly lower in women with 0 CL compared to 1 CL, both at 11 weeks (PI: 1.53, 95% CI [1.38–1.69] vs 1.72 [1.65–1.79], *P* = 0.026; RI: 0.69, [0.66–0.73] vs 0.73 [0.72–0.75], *P* = 0.034) and at 22 weeks gestational age (PI: 0.64 [0.57–0.72] vs 0.81 [0.78–0.85], *P* < 0.001; RI: 0.44 [0.41–0.46] vs 0.51 [0.50–0.53], *P* < 0.001). In pregnancies with >1 CL, uterine artery indices were comparable to the 1 CL group, except for a slightly higher RI at 22 weeks (0.54 [0.52–0.55], *P* = 0.011). Restricting the analyses to only pregnancies conceived using ARTs did not change the observed directions of the effects.

**LIMITATIONS, REASONS FOR CAUTION:**

This study was conducted in a tertiary hospital setting, which may limit generalizability to other populations. Details on luteal support were incomplete, and the corpus luteum number was inferred based on the mode of conception, which could introduce confounding by indication.

**WIDER IMPLICATIONS OF THE FINDINGS:**

These results align with previous literature and provide robust evidence from a large cohort, adjusting for confounders. Notably, uterine artery models were additionally adjusted for the observed differences in mean arterial pressure. However, despite this adjustment, the differences in uterine artery indices between CL groups persisted, indicating that these cannot be explained by the higher mean arterial pressure and suggesting the involvement of distinct vascular mechanisms. The observed differences in circulatory adaptation to pregnancy between conceptions with corpus luteum numbers may underlie the higher incidence of hypertensive disorders of pregnancy after conception without a corpus luteum. Additionally, these insights further support the preference for certain ARTs, where feasible, to optimize maternal and neonatal outcomes.

**STUDY FUNDING/COMPETING INTEREST(S):**

This research was funded by the Departments of Obstetrics and Gynaecology and Internal Medicine of the Erasmus MC, University Medical Center, Rotterdam, the Netherlands. The authors declare no competing interests.

**TRIAL REGISTRATION NUMBER:**

This study is registered at the Dutch Trial Register (NTR6854).

## Introduction

The use of ARTs has increased significantly in recent decades, offering many subfertile couples the possibility of achieving pregnancy. However, pregnancies conceived through ART are associated with slightly increased risks for both maternal and neonatal complications compared to spontaneously conceived pregnancies (Qin *et al.*, 2016; Conrad *et al.*, 2024). Several factors inherent to the ART process may contribute to these risks, such as ovarian stimulation protocols, oocyte handling, laboratory techniques, and culture media (Santos *et al.*, 2010; Youssef *et al.*, 2015). Despite the identification of these factors, the pathophysiological mechanisms associating ART protocols with certain pregnancy complications remain poorly understood. In this context, the number of corpora lutea (CL) present during early pregnancy, determined by the specific ART protocol, has emerged as a potential factor influencing differences in pregnancy outcomes (Conrad and Baker, 2013).

Previous studies have demonstrated significant variation in the incidence of hypertensive disorders of pregnancy (HDP) and birthweight outcomes depending on CL number. Specifically, pregnancies without a CL, conceived through programmed (artificial-cycle) frozen embryo transfer (AC-FET), are associated with nearly double the risk of preeclampsia and all HDP, compared to the physiological state of one CL during natural pregnancy (Conrad *et al.*, 2022, 2024; Koerts *et al.*, 2025). Moreover, evidence suggests that pregnancies after ovarian stimulation treatment and fresh embryo transfer, and thus conceived in the presence of multiple CL, have an increased risk of neonates born small-for-gestational-age (SGA) compared to the physiological state of one CL (Elias *et al.*, 2020; Landsverk *et al.*, 2023; Conrad *et al.*, 2024).

To explain the relationship with the number of CLs, several mechanisms have been proposed. A key hypothesis centres on the hormonal milieu associated with the presence or absence of CL. The CL is known to secrete critical hormones such as relaxin, prorenin, and progesterone, which are involved in the maternal adaptation to pregnancy (Chapman *et al.*, 1998; Conrad and Baker, 2013). Indeed, our previous work has demonstrated reduced activity of the renin–angiotensin–aldosterone system (RAAS) in pregnancies lacking a CL (Derkx *et al.*, 1987; Wiegel *et al.*, 2020, 2021a). This altered hormonal environment is suggested to disrupt maternal circulatory adaptation to pregnancy, resulting in abnormal blood pressure regulation and altered uterine artery blood flow.

Two studies have investigated the relationship between CL number and maternal blood pressure across all trimesters of pregnancy, reporting contrasting results (von Versen-Hoynck *et al.*, 2020; Wiegel *et al.*, 2021a). Differences in uterine artery Doppler indices, particularly pulsatility index (PI), between ART protocols have been more frequently studied, with lower PI reported in pregnancies with no CL compared to natural conceptions or fresh embryo transfers (Inversetti *et al.*, 2018; Choux *et al.*, 2019; Cavoretto *et al.*, 2020a,b; Wiegel *et al.*, 2021a). However, the studies lacked comparisons across all three CL groups (0, 1, and >1 CL), and no study has systematically assessed these differences throughout pregnancy.

By including a larger cohort and adjusting for potential confounders, this study aims to provide improved statistical power and a more accurate understanding of the relationship between CL number and maternal circulatory adaptation, focusing on blood pressure trajectories and uterine artery Doppler indices from early pregnancy onwards. Understanding these associations may provide insights into the pathophysiological mechanisms underlying the increased risk of hypertensive disorders in pregnancies conceived through ART and contribute to optimizing ART protocols and improving maternal and neonatal outcomes.

## Materials and methods

### Participant selection

This study utilizes data from the Rotterdam Periconception Cohort, a prospective observational study conducted at the Erasmus MC, University Medical Center, Rotterdam, the Netherlands (Steegers-Theunissen *et al.*, 2016; Rousian *et al.*, 2021). Women aged 18 or older with a singleton intrauterine pregnancy and who were proficient in Dutch were eligible for inclusion. Participants were enrolled before 10 weeks of gestation at the outpatient clinic. To ensure data independence, only the first pregnancy was included for women who had multiple pregnancies. Exclusion criteria for this study were ovulation induction, structural foetal anomalies, miscarriage, or unknown mode of conception. The study was approved by the Central Committee on Research in The Hague and the Medical Ethical Committee of Erasmus Medical Center (MEC-2004-227), with written informed consent obtained from all participants.

### ART treatment protocols

All ART treatments were conducted in a single centre, ensuring uniform procedures. The protocols for ovarian stimulation, oocyte collection, IVF, ICSI, and embryo assessment have been outlined previously (Hohmann *et al.*, 2003; Heijnen *et al.*, 2007). Ovarian stimulation involved recombinant FSH or human menopausal gonadotropin, paired with either a gonadotropin-releasing hormone agonist or antagonist. Final oocyte maturation was triggered by a single dose of human chorionic gonadotropin. Fertilization was performed through standard IVF and ICSI procedures (Heijnen *et al.*, 2007). The procedures used for embryo selection and transfer were described previously (Koerts *et al.*, 2025). Details on luteal support in different ART protocols are outlined below.

In the case of fresh embryo transfer, the luteal support protocols have evolved over the years. From the start of the study cohort in 2010, Utrogestan (progesterone) 200 mg was given vaginally for 12 days, starting 3 days after oocyte collection. Starting in June 2018, Rekovelle (recombinant FSH) was used as stimulating medication. From then on, Utrogestan was given for 15 days, starting the day after oocyte collection. Starting in March 2024, Utrogestan is now given for 7 weeks, starting on the evening after oocyte collection. In patients with peanut or soy allergies, Duphaston 10 mg three times per day or Crinone progesterone gel one time per day was used instead of Utrogestan. Patients diagnosed with hypothalamic or functional anovulation received Pregnyl (human chorionic gonadotropin) 1500 units on Days 2, 4, and 6 after oocyte collection.

In the case of natural cycle frozen embryo transfer (NC-FET), no luteal support was given. In the case of frozen embryo transfer in an artificial cycle, Progynova was given in increasing daily doses starting at 1 mg until 3 mg on the day of oocyte collection. Utrogestan was given starting 5 days before embryo transfer until the start of the 12th week of gestation. Afterwards, 2 mg Progynova was given two times per day.

### Data collection

Data on maternal characteristics, fertility treatment, pregnancy outcomes, and blood pressure measurements, taken in clinical settings from the first trimester to the first week postpartum using manual or automated devices, were collected from electronic health records and study databases.

At least four study visits were scheduled at specific weeks of gestation, including two in the first trimester (at 7, 9, 11, and/or 13 weeks of gestation) and two in the second and third trimester (at 22–24 weeks and 30–32 weeks of gestation). During the initial visit, systolic blood pressure (SBP) and diastolic blood pressure (DBP) were measured in mmHg by a nurse or physician using a manual cuff and stethoscope on the left arm, with the patient seated. Mean arterial pressure (MAP) was calculated as: (SBP + 2 × DBP) / 3.

For patients included from December 2016 onwards, bilateral measurements of the uterine artery PI and resistance index (RI) were obtained by trained sonographers at 7, 9, 11, 13, 22, and 30 weeks of gestation. Transvaginal ultrasound was performed in the first trimester and transabdominal ultrasound in the second and third trimesters using the Voluson Expert E8 or E10 ultrasound system with a 6–12 MHz transvaginal probe or transabdominal 3D probe (GE Healthcare, Austria) using standard power Doppler settings. At each time point, three measurements were taken on both the right and left sides, and the mean values of each side were used for analysis. Uterine artery notching, defined as a brief interruption or drop in velocity in the early diastolic waveform on Doppler ultrasound, was assessed at each study visit. The presence of a notch was recorded for each uterine artery (left and right), and persistent notching was defined as the presence of a notch across multiple visits during pregnancy.

Gestational age (GA) in natural pregnancies was determined from the first day of the last menstrual period (LMP) for women with regular cycles (21–35 days). For pregnancies conceived via IVF or ICSI, GA was calculated based on the oocyte retrieval day plus 14 days. In pregnancies from frozen embryo transfers, GA was calculated as the embryo transfer date plus 17 or 19 days, depending on the stage of embryo transfer. If there was a discrepancy of more than 6 days between the GA based on LMP and the crown–rump length (CRL) measurement, or if cycles were irregular or the LMP was unknown, GA was based on CRL.

### Study groups

Consistent with our previous research, pregnancies were categorized by the number of CL present at implantation, determined by the mode of conception (Wiegel *et al.*, 2020). The reference group, defined as 1 CL, included natural conceptions, intrauterine inseminations (IUI), and NC-FET. The 0 CL group included pregnancies conceived through AC-FET, while the >1 CL group involved pregnancies after ovarian stimulation treatment and fresh embryo transfer.

### Statistical analysis

Exposure categories were defined based on the number of CL (0, 1, or >1 CL). When multiple blood pressure measurements were available for a participant during the same visit, the mean of these measurements was calculated. Baseline characteristics, including pregnancy outcomes, were summarized as mean (SD), median (Q1, Q3), or n (%) and compared across groups using *t*-tests, Mann–Whitney *U*-tests, or Chi-square tests as appropriate. Pregnancy outcomes have been studied previously in the same cohort (Koerts *et al.*, 2025). Uterine artery indices were log-transformed to approximate a normal distribution. Linear mixed-effects regression models were used to assess blood pressure trajectories and changes in the uterine artery PI and RI. An interaction term was included in the uterine artery models to account for potential differences in PI and RI trajectories across CL groups. Additionally, a nested hierarchical model was developed, incorporating side (left or right) as a covariate. Associations between the CL group and outcomes were first examined with an unadjusted model accounting only for repeated measurements within each patient over gestation. A second, adjusted model controlled for maternal age, nulliparity, body mass index, smoking status and pre-existing hypertension, based on both population characteristics and relevant literature. For uterine artery indices, a third model was further adjusted for the observed differences in MAP. Moreover, the presence of uterine artery notches was analysed at each study visit. Sensitivity analyses were performed on ART pregnancies only to address potential confounding due to baseline differences between natural 1 CL pregnancies and NC-FET pregnancies. A separate sensitivity analysis was performed excluding pregnancies involving oocyte donation to account for potential additional confounding factors. After observing the unequal distribution of pregnancies conceived after ICSI across CL groups, a post-hoc analysis was conducted by introducing an interaction term into the model to evaluate potential effect modification. Model comparison was performed using likelihood ratio tests and analysis of variance. All analyses were conducted using R software version 4.3.2.

## Results

### Study population

**Figure 1.**
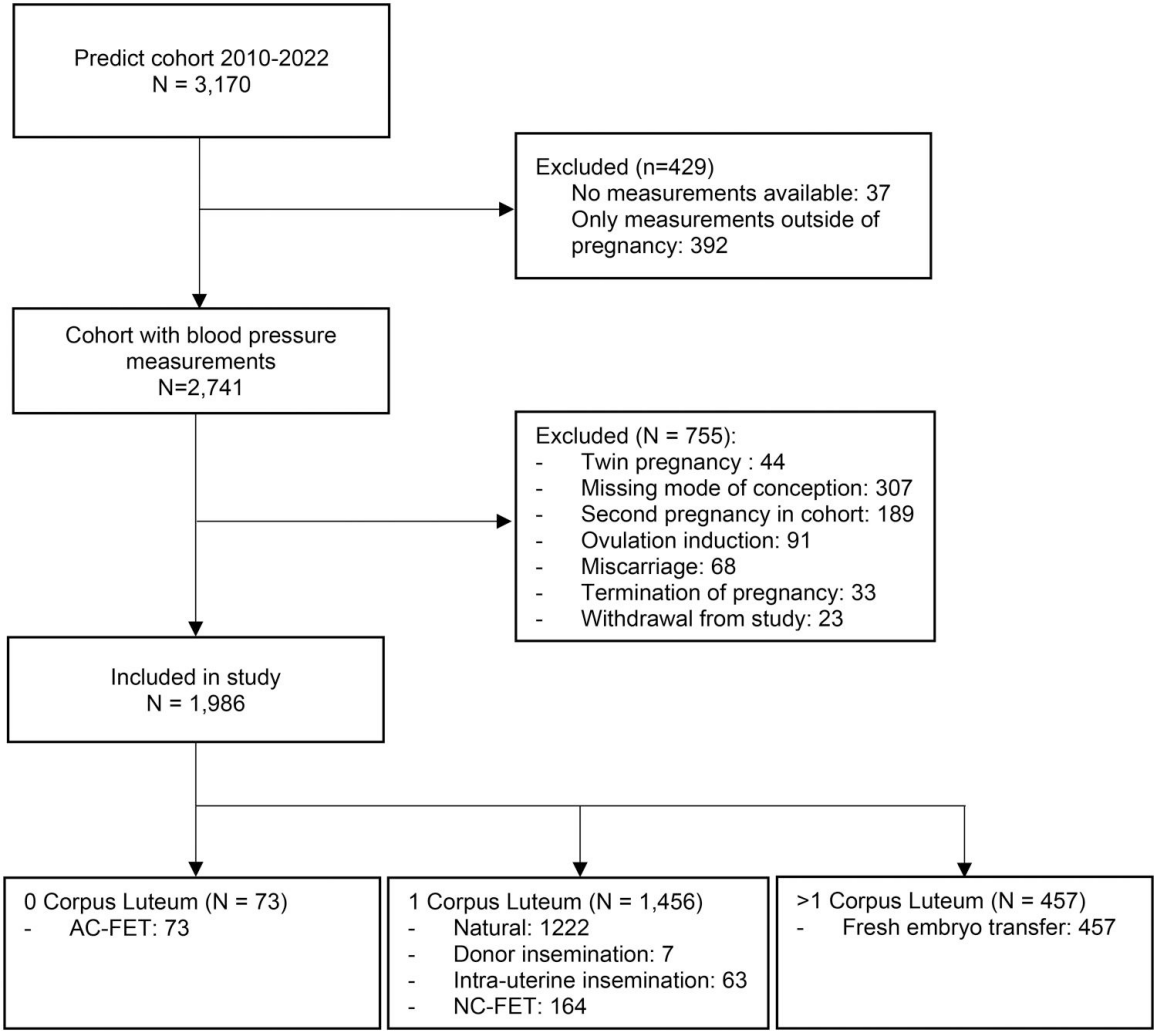
Flowchart of inclusion.

During the study period, a total of 3170 pregnant women met the eligibility criteria for inclusion. The selection process of participants is outlined in the flowchart in Fig. 1. Following the exclusion criteria 1986 patients were included in the final analysis. These could be categorized into the three CL groups: 1456 women with 1 CL, 457 women with >1 CL, and 73 women with 0 CL, including 23 with oocyte donation. Additional information on patient characteristics and mode of conception is shown in Supplementary Table S1.

### Baseline characteristics and pregnancy outcomes

The baseline characteristics across the different CL groups are summarized in Table 1. Mean maternal age was significantly higher in the 0 CL and >1 CL groups compared to the 1 CL group (33.5 and 33.1 years vs 31.7 years, respectively; *P* < 0.001). The median maternal BMI was lowest in women with 0 CL compared to those with >1 CL (23.7 kg/m^2^ vs 24.6 kg/m^2^) and 1 CL (25.3 kg/m^2^). The proportion of women who smoked or consumed alcohol within 3 months prior to conception was significantly higher in the 1 CL group compared to both the 0 CL and >1 CL groups.

**Table 1.** Baseline and clinical characteristics and pregnancy outcomes of the study population stratified by corpus luteum number.

	0 CL	>1 CL	1 CL	*P*
n	73	457	1456	
Maternal age, years (SD)	33.5 (5.86)	33.1 (4.39)	31.7 (4.69)	**<0.001**
Missing	0 (0.0)	0 (0.0)	0 (0.0)	
Paternal age, years (SD)	35.26 (5.39)	36.38 (6.56)	34.28 (5.84)	**<0.001**
Missing	4 (5.5)	19 (4.2)	137 (9.4)	
Ethnicity, n (%)				**<0.001**
Dutch	57 (78.1)	353 (77.2)	872 (59.9)	
Western	3 (4.1)	27 (5.9)	44 (3.0)	
Non-western	7 (9.6)	53 (11.6)	198 (13.6)	
Missing	6 (8.2)	24 (5.3)	342 (23.5)	
Education level, n (%)				**<0.001**
Low	7 (9.6)	30 (6.6)	100 (6.9)	
Medium	25 (34.2)	159 (34.8)	405 (27.8)	
High	35 (47.9)	243 (53.2)	609 (41.8)	
Missing	6 (8.2)	25 (5.5)	342 (23.5)	
Maternal BMI, kg/m^2^ [Q1, Q3]	23.7 [21.4, 29.3]	24.6 [22.0, 28.0]	25.3 [22.7, 29.4]	**<0.001**
Missing	0 (0.0)	3 (0.7)	19 (1.3)	
PCOS, n (%)	35 (47.9)	83 (18.2)	154 (10.6)	**<0.001**
Missing	5 (6.8)	10 (2.2)	1288 (88.5)	
POI, n (%)	13 (17.8)	4 (0.9)	8 (0.5)	**<0.001**
Missing	2 (2.7)	1 (0.2)	318 (21.8)	
Pre-existing hypertension, n (%)	5 (6.8)	7 (1.5)	69 (4.7)	**0.008**
Missing	1 (1.4)	19 (4.2)	38 (2.6)	
Pre-existing diabetes, n (%)				
Type 1	0 (0.0)	1 (0.2)	15 (1.0)	**<0.001**
Type 2	1 (1.4)	0 (0.0)	10 (0.7)	**<0.001**
Missing	5 (6.8)	64 (14.0)	108 (7.4)	
Nulliparity, n (%)	49 (67.1)	340 (74.4)	637 (43.8)	**<0.001**
Missing	2 (2.7)	24 (5.3)	45 (3.1)	
History of recurrent miscarriages, n (%)	3 (6.7)	12 (3.8)	46 (7.3)	0.114
Missing	28 (38.4)	145 (31.7)	827 (56.8)	
Preeclampsia in a previous pregnancy, n (%)	0 (0.0)	2 (0.4)	71 (6.0)	**<0.001**
Maternal smoking—preconceptional (%)	2 (2.7)	41 (9.0)	187 (12.8)	**<0.001**
Missing	6 (8.2)	24 (5.3)	348 (23.9)	
Maternal alcohol use—preconceptional, n (%)	18 (24.7)	74 (16.2)	378 (26.0)	**<0.001**
Missing	6 (8.2)	25 (5.5)	347 (23.8)	
Folic acid supplement use, n (%)	67 (100.0)	432 (99.8)	1088 (97.7)	**<0.001**
Missing	6 (8.2)	24 (5.3)	342 (23.5)	
Mode of conception, n (%)				**<0.001**
Natural	0 (0.0)	0 (0.0)	1222 (83.9)	
IUI	0 (0.0)	0 (0.0)	70 (4.8)	
IVF	47 (64.4)	174 (38.1)	69 (4.7)	
IVF + ICSI	23 (31.5)	283 (61.9)	93 (6.4)	
IVF, with or without ICSI unknown	3 (4.1)	0 (0.0)	2 (0.1)	
Hypertensive disorders of pregnancy, n (%)	10 (13.7)	34 (7.4)	165 (11.3)	0.078
Missing	1 (1.4)	19 (4.2)	43 (3.0)	
Preeclampsia, n (%)	7 (9.6)	10 (2.3)	80 (5.6)	**0.007**
	1 (1.4)	18 (3.9)	38 (2.6)	
Early-onset preeclampsia (<34 weeks GA), n (% of preeclampsia)	3 (42.9)	3 (30.0)	25 (31.3)	0.912
Gestational diabetes, n (%)	13 (17.8)	36 (7.9)	127 (8.7)	**0.009**
Missing	1 (1.4)	22 (4.8)	39 (2.7)	
SGA (birthweight <10th percentile), n (%)	6 (8.2)	67 (14.7)	197 (13.5)	0.439
LGA (birthweight >90th percentile), n (%)	6 (8.2)	31 (6.8)	143 (9.8)	0.286
Birthweight missing	4 (5.5)	29 (6.3)	72 (4.9)	

Continuous variables are reported as mean (SD) or median [Q1, Q3]. Categorical variables are reported as n (%), based on the number of non-missing observations. Pregnancy outcomes were partially reported in previously published data (Koerts *et al.*, 2025).

CL, corpus luteum; GA, gestational age; PCOS, polycystic ovarian syndrome; POI, premature ovarian insufficiency; SGA, small-for-gestational-age; LGA, large-for-gestational-age. Bold values indicate statistical significance (*P* < 0.05).

The number of pregnancies conceived after ICSI differed across CL groups, with the highest proportion in the >1 CL group (61.9%). Polycystic ovary syndrome (PCOS) was most prevalent in the 0 CL group (47.9%), as was premature ovarian insufficiency (17.8%), compared to the >1 CL and 1 CL groups, although a high number of missing records in the >1 CL and 1 CL groups may affect direct comparisons. The percentage of nulliparous women was significantly higher in women with >1 CL (74.4%) compared to those with 0 CL and 1 CL (67.1% and 43.8%, respectively, *P* < 0.001). In multiparous women, preeclampsia in a previous pregnancy occurred more often in the 1 CL group. Pre-existing hypertension was observed less frequently in the >1 CL group compared to the 0 CL and 1 CL groups (1.5% vs 6.8% and 4.7%, respectively).

The occurrence of HDP varied across the CL groups, with the highest rate observed in the 0 CL group (13.7%, *P* = 0.078) (Table 1). Preeclampsia was significantly more common in the 0 CL group (9.6%) compared to both the 1 CL and >1 CL groups (5.6% and 2.3%, *P* = 0.007), but no difference in the proportion of early- or late-onset preeclampsia cases was observed. Additionally, the 0 CL group had a significantly higher prevalence of gestational diabetes compared to 1 CL and >1 CL (17.8% vs 8.7% and 7.9%, *P* = 0.009). There were no significant differences for rates of SGA and large-for-gestational-age neonates. Note that some of these findings have been previously published, since the current study was performed within a subgroup of the same cohort (Koerts *et al.*, 2025).

### Blood pressure patterns

A total of 8595 unique measurements were collected, with 2829 in the first trimester, 2372 in the second trimester, and 3394 in the third trimester of pregnancy. These measurements corresponded to 1777 unique patients in the first trimester, 635 in the second trimester, and 607 in the third trimester.

**Figure 2.**
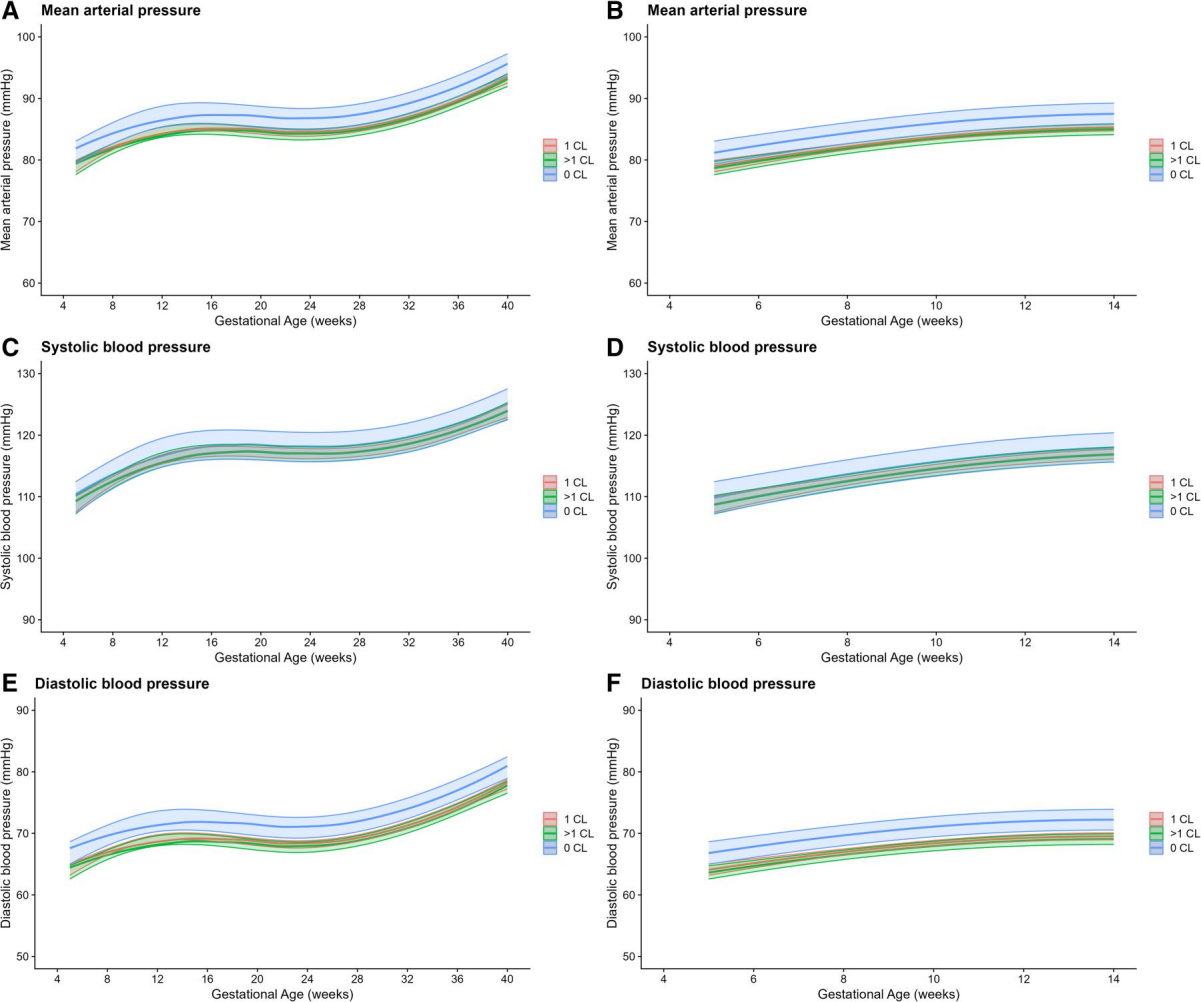
**Blood pressure trajectories throughout pregnancy by corpus luteum number.** Mean arterial pressure, systolic, and diastolic blood pressure throughout pregnancy (**A**, **C**, and **E**) and in the first trimester only (**B**, **D**, and **F**) in women with 0 CL, 1 CL, and >1 CL, after adjusting for gestational age, maternal age at conception, BMI, nulliparity, smoking in the periconception period, and pre-existing hypertension (Model 2). CL, corpus luteum.

At 9 weeks, MAP was 82.5 mmHg (95% CI: 82.0–82.9) in the 1 CL group, compared to 84.8 mmHg (95% CI: 83.0–86.6, *P* = 0.008) in the 0 CL group and 81.4 (95% CI: 80.6–82.1, *P* = 0.020) in the >1 CL group. As there was no significant interaction between GA and CL group, the trajectory of MAP over pregnancy was similar across groups, with observed differences reflecting a consistent offset. The unadjusted MAP during pregnancy was significantly higher in women with 0 CL (β 2.54, 95% CI: 0.68–4.41, *P* = 0.008), and lower in women with >1 CL (β −1.06 mmHg, 95% CI: −1.93 to −0.19, *P* = 0.017) compared to those with 1 CL at any given time during pregnancy (Table 2, Fig. 2). After adjusting, MAP remained higher in women with 0 CL (β 2.19, 95% CI: 0.43–3.95, *P* = 0.015), but was comparable between those with 1 and >1 CL. Similar effects were observed for DBP but not for SBP.

**Table 2.** Effect estimates of linear mixed models for difference in blood pressure and for 0 CL and >1 CL.

		0 CL		>1 CL	
		Beta (95% CI)	*P*-value	Beta (95% CI)	*P*-value
All trimesters					
Systolic blood pressure	Model 1	1.45 (−1.05 to 3.95)	0.257	−1.05 (−2.22 to 0.12)	0.079
	Model 2	1.08 (−1.33 to 3.48)	0.381	−0.08 (−1.32 to 1.15)	0.894
Diastolic blood pressure	Model 1	2.97 (1.19–4.74)	**0.001**	−1.12 (−1.95 to −0.28)	**0.009**
	Model 2	2.70 (1.01–4.39)	**0.002**	−0.46 (−1.33 to 0.41)	0.302
Mean arterial pressure	Model 1	2.54 (0.68–4.41)	**0.008**	−1.06 (−1.93 to −0.19)	**0.017**
	Model 2	2.19 (0.43–3.95)	**0.015**	−0.35 (−1.22 to 0.53)	0.438

1 CL as reference group. Model 1: adjusted for gestational age. Model 2: adjusted for gestational age, maternal age at conception, BMI, nulliparity, smoking in periconception period, and pre-existing hypertension.

CL, corpus luteum. Bold values indicate statistical significance (*P* < 0.05).

### Uterine artery Doppler

Uterine artery measurements were available for a subgroup of 624 patients: n = 32 in the 0 CL group, n = 398 in the 1 CL group, and n = 194 in the >1 CL group. A total of 2473 unique measurements were collected, with 1418 in the first trimester, specifically 119 in Week 7, 580 in Week 9, 534 in Week 11, and 185 in Week 13. In the second and third trimester, 550 and 505 measurements were performed, respectively. These measurements corresponded to 608 unique patients in the first trimester, 550 in the second trimester, and 505 in the third trimester. A total of 356 patients completed all four scheduled study visits.

**Figure 3.**
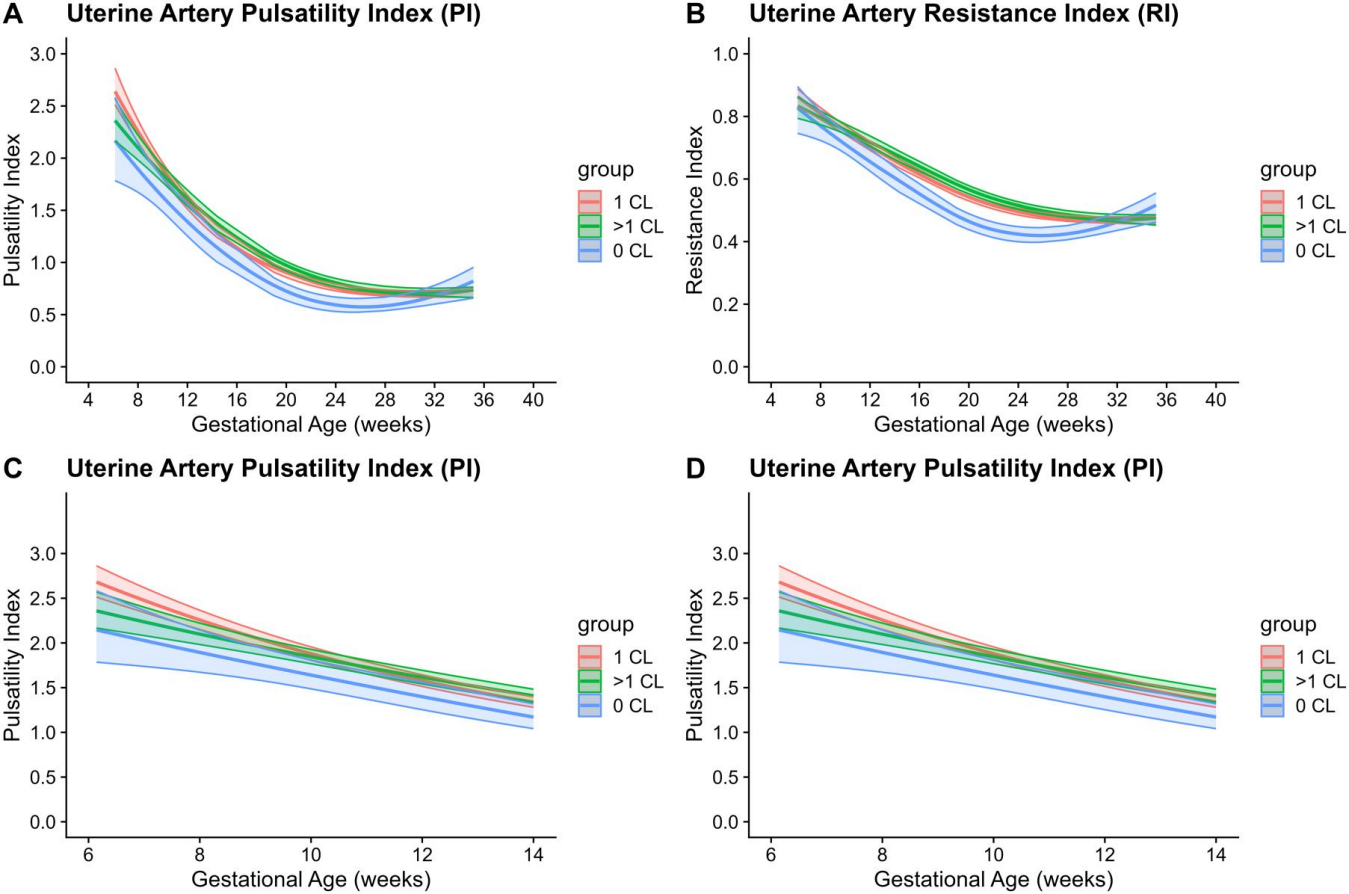
**Uterine artery Doppler indices throughout pregnancy by corpus luteum number.** Uterine artery pulsatility and resistance indices throughout pregnancy (**A** and **B**) and in the first trimester only (**C** and **D**) in women with 0 CL, 1 CL, and >1 CL, after adjusting for gestational age, maternal age at conception, BMI, nulliparity, smoking in the periconception period, and pre-existing hypertension (Model 2). CL, corpus luteum.

After adjusting for maternal and pregnancy-related characteristics (including maternal age at conception, BMI, nulliparity, smoking, and pre-existing hypertension), uterine artery PI and RI were lower in pregnancies with 0 CL compared to those with 1 CL in the first and second trimesters of pregnancy (Table 3, Fig. 3). In pregnancies with >1 CL, uterine artery indices were comparable to the 1 CL group in the first and third trimester, but slightly higher in the second trimester (RI: 0.54 (95% CI: 0.52–0.55) vs 0.51 (95% CI: 0.50–0.53), *P*-value = 0.009, Table 3, Fig. 3).

**Table 3.** Uterine artery Doppler indices for corpus luteum groups.

		0 CL			>1 CL			1 CL	
		Mean	95% CI	*P*-value	Mean	95% CI	*P*-value	Mean	95% CI
7 weeks	UtA PI	2.03	1.74–2.36	**0.010**	2.24	2.08–2.40	**0.026**	2.48	2.34–2.62
	UtA RI	0.79	0.74–0.86	0.209	0.81	0.78–0.84	0.217	0.84	0.81–0.86
9 weeks	UtA PI	1.77	1.59–1.96	**0.005**	1.97	1.88–2.06	0.170	2.06	1.97–2.15
	UtA RI	0.74	0.70–0.78	0.058	0.78	0.76–0.80	0.703	0.78	0.77–0.80
11 weeks	UtA PI	1.52	1.37–1.68	**0.020**	1.73	1.65–1.81	0.871	1.72	1.65–1.79
	UtA RI	0.69	0.65–0.72	**0.025**	0.74	0.72–0.76	0.384	0.73	0.72–0.75
22 weeks	UtA PI	0.63	0.57–0.71	**<0.001**	0.87	0.83–0.91	**0.039**	0.81	0.78–0.85
	UtA RI	0.44	0.41–0.46	**<0.001**	0.54	0.52–0.55	**0.009**	0.51	0.50–0.53
32 weeks	UtA PI	0.68	0.60–0.78	0.781	0.71	0.68–0.75	0.492	0.72	0.69–0.74
	UtA RI	0.46	0.44–0.50	0.812	0.47	0.46–0.49	0.433	0.47	0.46–0.48

Based on adjusted linear mixed models, side = right, no smoking, no pre-existing hypertension, nulliparous, mean BMI, and mean age. 1 CL as reference group. Adjusted for maternal age at conception, BMI, nulliparity, smoking in periconception period, and pre-existing hypertension.

CL, corpus luteum; PI, pulsatility index; RI, resistance index. Bold values indicate statistical significance (*P* < 0.05).

To evaluate the effect of MAP as investigated in our models (Table 2, Fig. 2), we included MAP as a potential confounder in the models for uterine artery PI and RI. This adjustment had minimal impact on most differences observed, as both remained significantly lower in women with 0 CL compared to those with 1 CL, both at 11 weeks (PI: 1.53, 95% CI [1.38–1.69] vs 1.72 [1.65–1.79], *P* = 0.026; RI: 0.69, 95% CI [0.66–0.73] vs 0.73 [0.72–0.75], *P* = 0.034) and at 22 weeks GA (PI: 0.64 [0.57–0.72] vs 0.81 [0.78–0.85], *P* < 0.001; RI: 0.44 [0.41–0.46] vs 0.51 [0.50–0.53], *P* < 0.001, Table 4). However, the significant difference in uterine artery PI between women with 1 and >1 CL at 22 weeks was no longer present, though it continued to show a trend towards a higher value compared to the 1 CL group. For reference, Supplementary Tables S2 and S3 present left and averaged uterine artery indices by GA and CL group.

**Table 4.** Uterine artery Doppler indices for corpus luteum groups, additionally adjusted for mean arterial pressure.

		0 CL			>1 CL			1 CL	
		Mean	95% CI	*P*-value	Mean	95% CI	*P*-value	Mean	95% CI
7 weeks	UtA PI	2.02	1.73–2.35	**0.012**	2.21	2.05–2.37	**0.025**	2.45	2.32–2.60
	UtA RI	0.79	0.73–0.85	0.237	0.81	0.78–0.84	0.187	0.83	0.81–0.85
9 weeks	UtA PI	1.77	1.59–1.96	**0.007**	1.95	1.86–2.05	0.143	2.05	1.97–2.14
	UtA RI	0.74	0.71–0.78	0.074	0.77	0.76–0.79	0.602	0.78	0.76–0.80
11 weeks	UtA PI	1.53	1.38–1.69	**0.026**	1.72	1.65–1.80	0.954	1.72	1.65–1.79
	UtA RI	0.69	0.66–0.73	**0.034**	0.74	0.72–0.76	0.464	0.73	0.72–0.75
22 weeks	UtA PI	0.64	0.57–0.72	**<0.001**	0.87	0.83–0.91	0.051	0.81	0.78–0.85
	UtA RI	0.44	0.41–0.46	**<0.001**	0.54	0.52–0.55	**0.013**	0.51	0.50–0.53
32 weeks	UtA PI	0.69	0.61–0.79	0.840	0.72	0.68–0.76	0.564	0.70	0.67–0.74
	UtA RI	0.47	0.44–0.51	0.892	0.48	0.46–0.49	0.523	0.47	0.46–0.48

Based on adjusted linear mixed models, side = right, no smoking, no pre-existing hypertension, nulliparous, mean BMI and mean age. 1 CL as reference group. Adjusted for maternal age at conception, BMI, nulliparity, smoking in periconception period, and pre-existing hypertension.

CL, corpus luteum; PI; pulsatility index; RI, resistance index. Bold values indicate statistical significance (*P* < 0.05).

We compared uterine artery notch presence between groups at all time points and examined whether first-trimester notches persisted into the second and third trimesters. A statistically significant difference in notch presence was observed only at 13 weeks, where the 1 CL group and >1 CL group exhibited higher rates of notching (Supplementary Table S4). At all other gestational stages examined, no significant differences were noted.

### Sensitivity analyses

In a sensitivity analysis restricted to ART pregnancies, both blood pressure and uterine artery results were consistent (Supplementary Tables S5 and S6). Excluding pregnancies with oocyte donation did not significantly alter the outcomes (data not shown). Furthermore, *post-hoc* analysis showed no significant effect modification of the higher proportion of ICSI pregnancies in the >1 CL group (data not shown).

## Discussion

### Principal findings

In this study, we examined the relationship between CL number and maternal circulatory adaptation in pregnancy, as assessed by longitudinal maternal blood pressure and uterine artery Doppler indices throughout pregnancy, using adjusted linear mixed model regression. Our analysis showed that MAP and DBP were significantly higher in women with 0 CL compared to those with 1 CL. Pregnancies with 0 CL exhibited significantly lower uterine artery PI and RI during the first and second trimesters compared to pregnancies with 1 CL. In contrast, pregnancies with >1 CL showed uterine artery indices that were comparable to the 1 CL group in the first and third trimesters but had a slightly higher PI in the second trimester (0.54 vs 0.52, *P*-value = 0.047). Adjusting the uterine artery models for MAP did not substantially alter these findings. Furthermore, there was no observed difference in SBP across CL groups, nor was there a distinct pattern of blood pressure change over the course of pregnancy. These findings suggest that the absence of CL affects both blood pressure and uterine vascular resistance in early to mid-pregnancy.

### Comparison with existing literature

Three studies have previously investigated the relationship between CL number and maternal blood pressure. Von Versen-Hoynck *et al.* (2019) reported a trend towards a higher 3–5 mmHg SBP, DBP and MAP in 0 CL compared to 1 CL pregnancies in the first and third trimesters, based on their supplementary data (von Versen-Hoynck *et al.*, 2019). Similar to our findings, von Versen-Hoynck (2020) observed no significant differences in blood pressure trajectories between CL groups in their unadjusted mixed model analysis, which included preconceptional, first, and third trimester measurements (von Versen-Hoynck *et al.*, 2020). Lastly, our previous study by Wiegel *et al.* (2021a,b) observed a lower MAP in the third trimester for 0 CL pregnancies, but this was based on unadjusted data from a smaller sample. Taken together, our study extends these findings by providing adjusted, longitudinal blood pressure trajectories by CL number in a larger cohort. Although a mid-pregnancy drop in blood pressure is often described, we observed a slight rise between Weeks 6 and 12 followed by stabilization until Week 24, which aligns with previous studies reporting more variable or absent mid-gestational declines, particularly in normotensive or low-risk populations (Nama *et al.*, 2011; Shen *et al.*, 2017).

The observed lower uterine artery PI in pregnancies with 0 CL also aligns with several prior studies, even though many did not directly compare based on CL groups (Inversetti *et al.*, 2018; Choux *et al.*, 2019; Cavoretto *et al.*, 2020a,b; Wiegel *et al.*, 2021a). However, the CL number can often be derived from the study design and the ART treatments that are compared, allowing us to relate their findings to our research focus. For uterine artery Doppler indices, our observation of lower uterine artery PI in 0 CL pregnancies aligns with studies by Inversetti *et al.* (2018) and Cavoretto *et al.* (2020b), who reported lower PI in donor oocyte pregnancies (typically 0 CL) compared to natural conceptions (Inversetti *et al.*, 2018; Cavoretto *et al.*, 2020b). Two other studies comparing pregnancies from AC-FET (0 CL) and fresh embryo transfer (>1 CL) further support our findings of a lower PI in pregnancies with 0 CL (Choux *et al.*, 2019; Cavoretto *et al.*, 2020a). However, these studies lacked comparisons with NC-FET and natural pregnancies (1 CL) and did not fully adjust for confounders. One other study investigated PI and RI at the start of progesterone supplementation and 12 weeks of gestation and found a steeper, though non-significant, decline in uterine artery PI and RI in the 0 CL (AC-FET) group compared to 1 CL (NC-FET), consistent with our findings (Lawrenz *et al.*, 2022). Our study by Wiegel *et al.* (2021a) similarly reported lower uterine artery PI and RI in AC-FET compared to natural and fresh embryo transfer pregnancies. The current study, using a larger, well-adjusted cohort, reinforces these findings across trimesters, providing more robust evidence of lower PI and RI in 0 CL pregnancies.

Uterine artery notching reflects increased resistance to blood flow in the uteroplacental circulation. A persistent notch is associated with impaired vascular remodelling and increased risk of placenta-related pregnancy outcomes (Cnossen *et al.*, 2008). Since CL number is also associated with these outcomes, e.g. SGA pregnancies, notching could be different between CL groups. While one previous study found differences in first trimester notching rates between fresh embryo transfer and natural pregnancies, we observed no significant differences between groups (Choux *et al.*, 2019). This suggests that CL number may primarily affect PI and RI rather than notching.

### Hypotheses and potential mechanisms

Evidence shows that pregnancies without a corpus luteum exhibit attenuated rises in progesterone, prorenin, and renin activity during early pregnancy, as well as the absence of circulating relaxin (Conrad *et al.*, 2019a; Wiegel *et al.*, 2020). These hormonal alterations could underlie the different vascular characteristics. In 0 CL pregnancies, reduced levels of relaxin may lead to reduced vascular relaxation and slightly elevated blood pressure (Conrad, 2011; Conrad *et al.*, 2022). This relative hypertension, combined with low prorenin levels due to the absent CL, appears to suppress the renin–angiotensin-aldosterone system (RAAS). Moreover, lower progesterone or oestrogen could also underlie this reduced RAAS activation (Komukai *et al.*, 2010; O'Donnell *et al.*, 2014; Wiegel *et al.*, 2021b). The resulting reduced RAAS activation likely contributes to insufficient plasma volume expansion, which is essential for normal pregnancy adaptation (Chapman *et al.*, 1998; Wiegel *et al.*, 2020, 2021b). This hypothesis is supported by a prior study showing blunted increases in left atrial size and persistent reduction in E wave velocity in 0 CL pregnancies, suggestive of reduced plasma volume expansion in this group (Conrad *et al.*, 2019b). Another study (Conrad *et al.*, 2019a) reported a steeper decline in plasma protein concentrations in the >1 CL group, possibly reflecting greater volume expansion; however, no such difference was observed between the 0 and 1 CL groups, and haemoglobin levels declined similarly across all groups. While these observations all support the proposed hypothesis of hormonal alterations underlying circulatory changes, direct evidence that restoring these hormones can reverse these changes remains lacking.

One previous study observed a positive correlation between serum relaxin and uterine artery RI at 10–12 weeks of gestation, which aligns with our finding of higher uterine artery RI in pregnancies with 1 CL or >1 CL compared to 0 CL pregnancies (Anumba *et al.*, 2009). However, the coexistence of higher MAP and lower uterine artery indices in women with 0 CL, thus in the absence of both relaxin and prorenin, remains complex and unclear. Adjusting the uterine artery models for MAP did not substantially alter the observed effects, suggesting that these measurements may not be directly connected.

The results might suggest the possibility of a compensatory decrease in uterine artery resistance to maintain adequate placental perfusion in 0 CL pregnancies. However, there is currently no evidence to support this hypothesis. One previous study observed that the increase in cardiac output during the first trimester, seen in spontaneous pregnancies (1 CL), is attenuated in 0 CL pregnancies (Conrad *et al.*, 2019b). Future studies could explore cardiac output differences between NC-FET (1 CL) and AC-FET pregnancies to eliminate confounding by natural vs ART conception. In addition, it would be valuable to investigate whether supplementation with CL-derived hormones can restore normal cardiovascular adaptation in 0 CL pregnancies. Additionally, one possible explanation for the observed lower uterine artery indices, as proposed by Conrad *et al.* (2024), could be greater than normal spiral artery remodelling in 0 CL pregnancies (Conrad *et al.*, 2024). However, this hypothesis has not yet been investigated.

### Strengths and limitations

The large cohort size of this study enhances statistical power, enabling us to adjust for key confounding factors such as maternal age, BMI, pre-existing hypertension, smoking status, and parity. All three CL groups, 0, 1, and >1 CL, were included within one analysis, providing a unique perspective on the role of CL number in pregnancy circulation. Finally, by including only ART-conceived pregnancies in a sensitivity analysis, we minimized variability from natural conception, strengthening our comparisons and reducing potential confounding.

Despite these strengths, there are limitations to consider. The study was conducted within a tertiary hospital setting, which may affect the generalizability of our findings to more diverse, low-risk populations. Additionally, we did not have detailed data on progesterone luteal support. Standard protocols differ across CL groups: fresh embryo transfers typically receive short-term vaginal progesterone supplementation, with additional choriogonadotropin in case of hypothalamic or functional anovulation, while NC-FET cycles do not receive luteal support. In contrast, AC-FET cycles received both progesterone and oestradiol supplementation until 12 weeks of gestation. We should also note here that the CL number was based on the conception mode rather than the ultrasound findings. Another potential limitation is confounding by indication, as fresh embryo transfers are typically attempted first and may selectively represent ‘successful’ pregnancies with favourable outcomes. If fresh transfer is unsuccessful, frozen transfer is often used, and this group may include pregnancies with underlying factors that initially contributed to unsuccessful fresh transfer, potentially impacting pregnancy outcomes. Furthermore, women without a natural cycle, like those with PCOS, are more likely to be in the 0 CL group, and studies have shown a higher risk of preeclampsia and elevated blood pressure in women with PCOS (Qin *et al.*, 2013, Valdimarsdottir *et al.*, 2024). Lastly, data on antihypertensive medication use during pregnancy or preconceptional blood pressure measurements were not available in this study. Therefore, we cannot rule out the possibility that the observed higher blood pressure in the 0 CL group may partly reflect pre-existing differences, as reported in prior studies (Conrad *et al.*, 2019b; Von Versen-Höynck *et al.*, 2019).

## Conclusion

In this study, we examined maternal blood pressure across pregnancies with 0, 1, and >1 corpus luteum (CL) throughout gestation. We found that women with 0 CL had significantly higher MAP and DBP compared to those with 1 CL, while no differences were observed between the 1 and >1 CL groups. Additionally, uterine artery Doppler indices showed that 0 CL pregnancies had lower PI and RI in the first and second trimesters compared to 1 CL pregnancies, even after adjusting for the higher MAP. These findings suggest distinct vascular adaptations in 0 CL pregnancies, potentially influenced by embryo transfer protocols and the absence of the CL.

## Supplementary Material

deaf181_Supplementary_Table_S1

deaf181_Supplementary_Table_S2

deaf181_Supplementary_Table_S3

deaf181_Supplementary_Table_S4

deaf181_Supplementary_Table_S5

deaf181_Supplementary_Table_S6

## Data Availability

The data underlying this article will be shared upon reasonable request to the corresponding author.

## References

[deaf181-B1] Anumba DO , El Gelany S, Elliott SL, Li TC. Serum relaxin levels are reduced in pregnant women with a history of recurrent miscarriage, and correlate with maternal uterine artery Doppler indices in first trimester. Eur J Obstet Gynecol Reprod Biol 2009;147:41–45. 19695764 10.1016/j.ejogrb.2009.07.008

[deaf181-B2] Cavoretto PI , Farina A, Gaeta G, Sigismondi C, Spinillo S, Casiero D, Pozzoni M, Vigano P, Papaleo E, Candiani M. Uterine artery Doppler in singleton pregnancies conceived after in-vitro fertilization or intracytoplasmic sperm injection with fresh vs frozen blastocyst transfer: longitudinal cohort study. Ultrasound Obstet Gynecol 2020a;56:603–610. 31909549 10.1002/uog.21969

[deaf181-B3] Cavoretto PI , Farina A, Miglio R, Zamagni G, Girardelli S, Vanni VS, Morano D, Spinillo S, Sartor F, Candiani M. Prospective longitudinal cohort study of uterine arteries Doppler in singleton pregnancies obtained by IVF/ICSI with oocyte donation or natural conception. Hum Reprod 2020b;35:2428–2438. 33099621 10.1093/humrep/deaa235

[deaf181-B4] Chapman AB , Abraham WT, Zamudio S, Coffin C, Merouani A, Young D, Johnson A, Osorio F, Goldberg C, Moore LG et al Temporal relationships between hormonal and hemodynamic changes in early human pregnancy. Kidney Int 1998;54:2056–2063. 9853271 10.1046/j.1523-1755.1998.00217.x

[deaf181-B5] Choux C , Ginod P, Barberet J, Rousseau T, Bruno C, Sagot P, Astruc K, Fauque P. Placental volume and other first-trimester outcomes: are there differences between fresh embryo transfer, frozen-thawed embryo transfer and natural conception? Reprod Biomed Online 2019;38:538–548. 30850320 10.1016/j.rbmo.2018.12.023

[deaf181-B6] Cnossen JS , Morris RK, ter Riet G, Mol BW, van der Post JA, Coomarasamy A, Zwinderman AH, Robson SC, Bindels PJ, Kleijnen J et al Use of uterine artery Doppler ultrasonography to predict pre-eclampsia and intrauterine growth restriction: a systematic review and bivariable meta-analysis. CMAJ 2008;178:701–711. 18332385 10.1503/cmaj.070430 PMC2263112

[deaf181-B7] Conrad KP. Emerging role of relaxin in the maternal adaptations to normal pregnancy: implications for preeclampsia. Semin Nephrol 2011;31:15–32. 21266262 10.1016/j.semnephrol.2010.10.003 PMC3381791

[deaf181-B8] Conrad KP , Baker VL. Corpus luteal contribution to maternal pregnancy physiology and outcomes in assisted reproductive technologies. Am J Physiol Regul Integr Comp Physiol 2013;304:R69–R72. 23100030 10.1152/ajpregu.00239.2012 PMC3543656

[deaf181-B9] Conrad KP , Graham GM, Chi YY, Zhai X, Li M, Williams RS, Rhoton-Vlasak A, Segal MS, Wood CE, Keller-Wood M. Potential influence of the corpus luteum on circulating reproductive and volume regulatory hormones, angiogenic and immunoregulatory factors in pregnant women. Am J Physiol Endocrinol Metab 2019a;317:E677–E685. 31408378 10.1152/ajpendo.00225.2019 PMC6842916

[deaf181-B10] Conrad KP , Petersen JW, Chi YY, Zhai X, Li M, Chiu KH, Liu J, Lingis MD, Williams RS, Rhoton-Vlasak A et al Maternal cardiovascular dysregulation during early pregnancy after in vitro fertilization cycles in the absence of a corpus luteum. Hypertension 2019b;74:705–715. 31352818 10.1161/HYPERTENSIONAHA.119.13015 PMC6687559

[deaf181-B11] Conrad KP , von Versen-Hoynck F, Baker VL. Potential role of the corpus luteum in maternal cardiovascular adaptation to pregnancy and preeclampsia risk. Am J Obstet Gynecol 2022;226:683–699. 34437863 10.1016/j.ajog.2021.08.018

[deaf181-B12] Conrad KP , von Versen-Hoynck F, Baker VL. Pathologic maternal and neonatal outcomes associated with programmed embryo transfer. J Assist Reprod Genet 2024;41:821–842. 38536594 10.1007/s10815-024-03041-9 PMC11052974

[deaf181-B13] Derkx FH , Alberda AT, Zeilmaker GH, Schalekamp MA. High concentrations of immunoreactive renin, prorenin and enzymatically-active renin in human ovarian follicular fluid. Br J Obstet Gynaecol 1987;94:4–9. 3545283 10.1111/j.1471-0528.1987.tb02243.x

[deaf181-B14] Elias FTS , Weber-Adrian D, Pudwell J, Carter J, Walker M, Gaudet L, Smith G, Velez MP. Neonatal outcomes in singleton pregnancies conceived by fresh or frozen embryo transfer compared to spontaneous conceptions: a systematic review and meta-analysis. Arch Gynecol Obstet 2020;302:31–45. 32445067 10.1007/s00404-020-05593-4 PMC7266861

[deaf181-B15] Heijnen EM , Eijkemans MJ, De Klerk C, Polinder S, Beckers NG, Klinkert ER, Broekmans FJ, Passchier J, Te Velde ER, Macklon NS et al A mild treatment strategy for in-vitro fertilisation: a randomised non-inferiority trial. Lancet 2007;369:743–749. 17336650 10.1016/S0140-6736(07)60360-2

[deaf181-B16] Hohmann FP , Macklon NS, Fauser BC. A randomized comparison of two ovarian stimulation protocols with gonadotropin-releasing hormone (GnRH) antagonist cotreatment for in vitro fertilization commencing recombinant follicle-stimulating hormone on cycle day 2 or 5 with the standard long GnRH agonist protocol. J Clin Endocrinol Metab 2003;88:166–173. 12519847 10.1210/jc.2002-020788

[deaf181-B17] Inversetti A , Mandia L, Candiani M, Cetin I, Larcher A, Savasi V, Papaleo E, Cavoretto P. Uterine artery Doppler pulsatility index at 11-38 weeks in ICSI pregnancies with egg donation. J Perinat Med 2018;46:21–27. 28186956 10.1515/jpm-2016-0180

[deaf181-B18] Koerts JJ , Voskamp LW, Rousian M, Steegers-Theunissen RPM, Wiegel RE. Impact of corpus luteum number on maternal pregnancy and birth outcomes: the Rotterdam Periconception Cohort. Fertil Steril 2025;123:1039–1050. 39644989 10.1016/j.fertnstert.2024.12.002

[deaf181-B19] Komukai K , Mochizuki S, Yoshimura M. Gender and the renin-angiotensin-aldosterone system. Fundam Clin Pharmacol 2010;24:687–698. 20608988 10.1111/j.1472-8206.2010.00854.x

[deaf181-B20] Landsverk E , Westvik-Johari K, Romundstad LB, Opdahl S. Birth size after embryo cryopreservation: larger by all measures? Hum Reprod 2023;38:1379–1389. 37178338 10.1093/humrep/dead094 PMC10320486

[deaf181-B21] Lawrenz B , Markova D, Melado L, Vitorino RL, Digma S, Samir S, Fatemi HM. Prospective observational comparison of arteria uterina blood flow between two frozen embryo transfer cycle regimens: natural cycle versus hormonal replacement cycle. Arch Gynecol Obstet 2022;306:2177–2185. 36123426 10.1007/s00404-022-06789-6

[deaf181-B22] Nama V , Antonios TF, Onwude J, Manyonda IT. Mid-trimester blood pressure drop in normal pregnancy: myth or reality? J Hypertens 2011;29:763–768. 21178781 10.1097/HJH.0b013e328342cb02

[deaf181-B23] O'Donnell E , Floras JS, Harvey PJ. Estrogen status and the renin angiotensin aldosterone system. Am J Physiol Regul Integr Comp Physiol 2014;307:R498–R500. 24944241 10.1152/ajpregu.00182.2014

[deaf181-B24] Qin J , Liu X, Sheng X, Wang H, Gao S. Assisted reproductive technology and the risk of pregnancy-related complications and adverse pregnancy outcomes in singleton pregnancies: a meta-analysis of cohort studies. Fertil Steril 2016;105:73–85.e71–76. 26453266 10.1016/j.fertnstert.2015.09.007

[deaf181-B25] Qin JZ , Pang LH, Li MJ, Fan XJ, Huang RD, Chen HY. Obstetric complications in women with polycystic ovary syndrome: a systematic review and meta-analysis. Reprod Biol Endocrinol 2013;11:56. 23800002 10.1186/1477-7827-11-56 PMC3737012

[deaf181-B26] Rousian M , Schoenmakers S, Eggink AJ, Gootjes DV, Koning AHJ, Koster MPH, Mulders A, Baart EB, Reiss IKM, Laven JSE et al Cohort Profile Update: the Rotterdam Periconceptional Cohort and embryonic and fetal measurements using 3D ultrasound and virtual reality techniques. Int J Epidemiol 2021;50:1426–1427l. 34097026 10.1093/ije/dyab030 PMC8580268

[deaf181-B27] Santos MA , Kuijk EW, Macklon NS. The impact of ovarian stimulation for IVF on the developing embryo. Reproduction 2010;139:23–34. 19710204 10.1530/REP-09-0187

[deaf181-B28] Shen M , Tan H, Zhou S, Smith GN, Walker MC, Wen SW. Trajectory of blood pressure change during pregnancy and the role of pre-gravid blood pressure: a functional data analysis approach. Sci Rep 2017;7:6227. 28740155 10.1038/s41598-017-06606-0 PMC5524922

[deaf181-B29] Steegers-Theunissen RP , Verheijden-Paulissen JJ, van Uitert EM, Wildhagen MF, Exalto N, Koning AH, Eggink AJ, Duvekot JJ, Laven JS, Tibboel D et al Cohort profile: The Rotterdam Periconceptional Cohort (Predict Study). Int J Epidemiol 2016;45:374–381. 26224071 10.1093/ije/dyv147

[deaf181-B30] Valdimarsdottir R , Vanky E, Elenis E, Lindstrom L, Junus K, Jonsson M, Sundstrom Poromaa I, Wikstrom AK. Polycystic ovary syndrome and risk of pre-eclampsia: a national register-based cohort study. BJOG 2024;131:985–995. 38082470 10.1111/1471-0528.17734

[deaf181-B31] von Versen-Höynck F , Hackl S, Selamet Tierney ES, Conrad KP, Baker VL, Winn VD. Maternal vascular health in pregnancy and postpartum after assisted reproduction. Hypertension 2020;75:549–560. 31838910 10.1161/HYPERTENSIONAHA.119.13779 PMC7491550

[deaf181-B32] von Versen-Höynck F , Narasimhan P, Selamet Tierney ES, Martinez N, Conrad KP, Baker VL, Winn VD. Absent or excessive corpus luteum number is associated with altered maternal vascular health in early pregnancy. Hypertension 2019;73:680–690. 30636549 10.1161/HYPERTENSIONAHA.118.12046 PMC6378337

[deaf181-B33] Wiegel RE , Jan Danser AH, Steegers-Theunissen RPM, Laven JSE, Willemsen SP, Baker VL, Steegers EAP, von Versen-Hoynck F. Determinants of maternal renin-angiotensin-aldosterone-system activation in early pregnancy: insights from 2 cohorts. J Clin Endocrinol Metab 2020;105:3505–3517. 32853347 10.1210/clinem/dgaa582 PMC7494245

[deaf181-B34] Wiegel RE , Karsten MJH, Reijnders IF, van Rossem L, Willemsen SP, Mulders A, Koning AHJ, Steegers EAP, Danser AHJ, Steegers-Theunissen RPM. Corpus luteum number and the maternal renin-angiotensin-aldosterone system as determinants of utero-placental (vascular) development: the Rotterdam Periconceptional Cohort. Reprod Biol Endocrinol 2021a;19:164. 34732224 10.1186/s12958-021-00843-9 PMC8567673

[deaf181-B35] Wiegel RE , von Versen-Hoynck F, Steegers-Theunissen RPM, Steegers EAP, Danser AHJ. Prorenin periconceptionally and in pregnancy: does it have a physiological role? Mol Cell Endocrinol 2021b;522:111118. 33340569 10.1016/j.mce.2020.111118

[deaf181-B36] Youssef MM , Mantikou E, van Wely M, Van der Veen F, Al-Inany HG, Repping S, Mastenbroek S. Culture media for human pre-implantation embryos in assisted reproductive technology cycles. Cochrane Database Syst Rev 2015;2015:CD007876. 26585317 10.1002/14651858.CD007876.pub2 PMC10657458

