# Supplementary material for: Corpus luteum number and maternal circulatory adaptation from early pregnancy onwards: the Rotterdam Periconception Cohort (Predict Study)
Source: Hum Reprod. 2025 Sep 16;40(11):2078–87. doi: 10.1093/humrep/deaf181 (PMC12584914; doi:10.1093/humrep/deaf181)
Supplement: deaf181_Supplementary_Table_S1 [file deaf181_supplementary_table_s1.pdf]

Human Reproduction, 2025, 1–1
https://doi.org/10.1093/humrep/deaf181
Supplementary Data

Supplementary Table S1. Baseline and clinical characteristics and pregnancy outcomes of the study population stratified by mode of conception.

	0 CL		>1 CL		1 CL	
	AC-FET without OD	AC-FET with OD	Fresh ET	Spontaneous	IUI	NC-FET
n	50	23	457	1224	70	162
Maternal age, years (SD)	31.6 (4.18)	37.7 (6.86)	33.1 (4.39)	31.4 (4.75)	33.9 (3.24)	33.1 (4.25)
Missing	0 (0.0)	0 (0.0)	0 (0.0)	0 (0.0)	0 (0.0)	0 (0.0)
Paternal age, years (SD)	33.7 (4.40)	38.6 (5.89)	36.4 (6.56)	33.9 (5.74)	37.2 (6.60)	35.9 (5.79)
Missing	3 (6.0)	1 (4.3)	19 (4.2)	108 (8.8)	17 (24.3)	12 (7.4)
Ethnicity, n (%)						
Dutch	38 (76.0)	19 (82.6)	353 (77.2)	710 (58.0)	51 (72.9)	111 (68.5)
Western	1 (2.0)	2 (8.7)	27 (5.9)	34 (2.8)	5 (7.1)	5 (3.1)
Non-western	6 (12.0)	1 (4.3)	53 (11.6)	150 (12.3)	11 (15.7)	37 (22.8)
Missing	5 (10.0)	1 (4.3)	24 (5.3)	330 (27.0)	3 (4.3)	9 (5.6)
Education level, n (%)						
Low	4 (8.0)	3 (13.0)	30 (6.6)	85 (6.9)	3 (4.3)	12 (7.4)
Medium	20 (40.0)	5 (21.7)	159 (34.8)	329 (26.9)	22 (31.4)	54 (33.3)
High	21 (42.0)	14 (60.9)	243 (53.2)	480 (39.2)	42 (60.0)	87 (53.7)
Missing	5 (10.0)	1 (4.3)	25 (5.5)	330 (27.0)	3 (4.3)	9 (5.6)
Maternal BMI, kg/m^2 [Q1, Q3]	24.7 [21.5, 29.4]	22.3 [21.0, 25.1]	24.6 [22.0, 28.0]	25.3 [22.7, 29.6]	25.8 [23.0, 29.6]	25.1 [22.5, 28.7]
Missing	0 (0.0)	0 (0.0)	3 (0.7)	15 (1.2)	3 (4.3)	1 (0.6)
PCOS, n (%)	32 (64.0)	1 (4.3)	83 (18.2)	3 (0.2)	0 (0.0)	11 (6.8)
Missing	1 (2.0)	4 (17.4)	10 (2.2)	1216 (99.3)	70 (100.0)	2 (1.2)
POI, n (%)	0 (0.0)	13 (56.5)	4 (0.9)	7 (0.6)	0 (0.0)	1 (0.6)
Missing	1 (2.0)	1 (4.3)	1 (0.2)	315 (25.7)	3 (4.3)	0 (0.0)
Pre-existing hypertension, n (%)	3 (6.0)	2 (8.7)	7 (1.5)	59 (4.8)	5 (7.1)	5 (3.1)
Missing	1 (2.0)	0 (0.0)	19 (4.2)	26 (2.1)	4 (5.7)	8 (4.9)
Pre-existing diabetes, n (%)						
Type 1	0 (0.0)	0 (0.0)	1 (0.2)	14 (1.1)	0 (0.0)	1 (0.6)
Type 2	0 (0.0)	1 (4.3)	0 (0.0)	10 (0.8)	0 (0.0)	0 (0.0)
Missing	5 (10.0)	0 (0.0)	64 (14.0)	76 (6.2)	12 (17.1)	20 (12.3)
Nulliparity, n (%)	29 (58.0)	13 (56.5)	174 (38.1)	889 (72.6)	30 (42.9)	89 (54.9)
Missing	0 (0.0)	0 (0.0)	7 (1.5)	22 (1.8)	1 (1.4)	4 (2.5)
History of recurrent miscarriages, n (%)	12 (3.8)	2 (20.0)	12 (3.8)	38 (8.3)	1 (2.1)	7 (5.6)
Missing	145 (31.7)	13 (56.6)	145 (31.7)	765 (62.6)	23 (32.9)	37 (22.8)
Preeclampsia in a previous pregnancy, n (%)	0 (0.0)	0 (0.0)	2 (0.4)	66 (5.4)	1 (1.4)	4 (2.5)
Missing	1 (2.0)	2 (8.7)	29 (6.3)	253 (20.7)	6 (8.6)	17 (10.5)
Maternal smoking—preconceptional (%)	2 (4.0)	0 (0.0)	41 (9.0)	163 (13.3)	3 (4.3)	21 (13.0)
Missing	5 (10.0)	1 (4.3)	24 (5.3)	335 (27.4)	4 (5.7)	9 (5.6)
Maternal alcohol use—preconceptional, n (%)	14 (28.0)	4 (17.4)	74 (16.2)	316 (25.8)	24 (34.3)	38 (23.5)
Missing	5 (10.0)	1 (4.3)	25 (5.5)	334 (27.3)	4 (5.7)	9 (5.6)
Folic acid supplement use, n (%)	45 (90.0)	22 (95.7)	432 (94.5)	869 (71.0)	66 (94.3)	153 (94.4)
Missing	5 (10.0)	1 (4.3)	24 (5.3)	329 (26.9)	4 (5.7)	9 (5.6)
Mode of conception, n (%)						
Natural	0 (0.0)	0 (0.0)	0 (0.0)	1224 (100.0)	0 (0.0)	0 (0.0)
IUI	0 (0.0)	0 (0.0)	0 (0.0)	0 (0.0)	70 (100.0)	0 (0.0)
IVF	31 (62.0)	16 (69.6)	174 (38.1)	0 (0.0)	0 (0.0)	69 (42.6)
IVF+ ICSI	16 (32.0)	7 (30.4)	283 (61.9)	0 (0.0)	0 (0.0)	93 (57.4)
IVF, with or without ICSI unknown	3 (6.0)	0 (0.0)	0 (0.0)	0 (0.0)	0 (0.0)	0 (0.0)
Hypertensive disorders of pregnancy, n (%)	6 (12.0)	4 (17.4)	34 (7.4)	142 (11.6)	7 (10.0)	16 (9.9)
Missing	1 (2.0)	0 (0.0)	19 (4.2)	30 (2.5)	4 (5.7)	9 (5.6)
Preeclampsia, n (%)	4 (8.0)	3 (13.0)	10 (2.2)	65 (5.3)	6 (8.6)	9 (5.6)
Missing	1 (2.0)	0 (0.0)	18 (3.9)	26 (2.1)	4 (5.7)	8 (4.9)
Early-onset preeclampsia (<34 weeks ga), n (% of preeclampsia)	2 (50.0)	1 (33.3)	3 (30.0)	21 (32.3)	2 (33.3)	2 (22.2)
Gestational diabetes, n (%)	10 (20.0)	3 (13.0)	36 (7.9)	102 (8.3)	8 (11.4)	17 (10.5)
Missing	1 (2.0)	0 (0.0)	22 (4.8)	27 (2.2)	4 (5.7)	8 (4.9)
SGA (birthweight <10th percentile), n (%)	1 (2.0)	5 (21.7)	67 (14.7)	173 (14.1)	10 (14.3)	14 (8.6)
LGA (birthweight >90th percentile), n (%)	4 (8.0)	2 (8.7)	31 (6.8)	119 (9.7)	6 (8.6)	18 (11.1)
Birthweight missing	4 (8.0)	0 (0.0)	29 (6.3)	53 (4.3)	5 (7.1)	14 (8.6)

Continuous variables are reported as mean (SD) or median [Q1, Q3]. Pregnancy outcomes were partially reported in previously published data (Koerts *et al.*, 2025). AC-FET, artificial cycle frozen embryo transfer; CL, corpus luteum; OD, oocyte donation; fresh ET, fresh embryo transfer; NC-FET, natural cycle frozen embryo transfer; GA, gestational age; PCOS, polycystic ovarian syndrome; POI, premature ovarian insufficiency; SGA, small-for-gestational-age; LGA, large-for-gestational-age.
