# Supplementary material for: Corpus luteum number and maternal circulatory adaptation from early pregnancy onwards: the Rotterdam Periconception Cohort (Predict Study)
Source: Hum Reprod. 2025 Sep 16;40(11):2078–87. doi: 10.1093/humrep/deaf181 (PMC12584914; doi:10.1093/humrep/deaf181)
Supplement: deaf181_Supplementary_Table_S2 [file deaf181_supplementary_table_s2.pdf]

OXFORD
human reproduction
Human Reproduction, 2025, 1–1
https://doi.org/10.1093/humrep/deaf181
Supplementary Data

Supplementary Table S2. Uterine artery Doppler indices for corpus luteum groups.

		0 CL			>1 CL			1 CL	
		**Mean**	**95% CI**	**P-value**	**Mean**	**95% CI**	**P-value**	**Mean**	**95% CI**
7 weeks	UtA PI	2.08	1.79–2.43	**0.010**	2.30	2.14–2.47	**0.026**	2.55	2.41–2.69
	UtA RI	0.81	0.75–0.87	0.209	0.83	0.80–0.85	0.217	0.85	0.83–0.87
9 weeks	UtA PI	1.82	1.63–2.02	**0.005**	2.02	1.93–2.12	0.170	2.12	2.03–2.21
	UtA RI	0.75	0.72–0.79	0.058	0.79	0.77–0.81	0.703	0.79	0.80–0.81
11 weeks	UtA PI	1.56	1.41–1.73	**0.020**	1.78	1.70–1.86	0.871	1.77	1.70–1.84
	UtA RI	0.70	0.66–0.73	**0.025**	0.75	0.74–0.77	0.385	0.74	0.73–0.76
22 weeks	UtA PI	0.65	0.58–0.73	**<0.001**	0.90	0.85–0.94	**0.039**	0.84	0.80–0.87
	UtA RI	0.44	0.42–0.47	**<0.001**	0.55	0.53–0.56	**0.009**	0.52	0.51–0.53
32 weeks	UtA PI	0.70	0.62–0.80	0.781	0.74	0.70–0.77	0.492	0.72	0.69–0.75
	UtA RI	0.47	0.44–0.50	0.816	0.48	0.47–0.49	0.433	0.48	0.47–0.49

Based on adjusted linear mixed models, side = left, no smoking, no pre-existing hypertension, nulliparous, mean BMI and mean age. 1 CL as reference group.
Adjusted for maternal age at conception, BMI, nulliparity, smoking in periconception period, and pre-existing hypertension.
CL, corpus luteum; PI, pulsatility index; RI, resistance index. Bold values indicate statistical significance ($P < 0.05$).
