# Supplementary material for: Corpus luteum number and maternal circulatory adaptation from early pregnancy onwards: the Rotterdam Periconception Cohort (Predict Study)
Source: Hum Reprod. 2025 Sep 16;40(11):2078–87. doi: 10.1093/humrep/deaf181 (PMC12584914; doi:10.1093/humrep/deaf181)
Supplement: deaf181_Supplementary_Table_S3 [file deaf181_supplementary_table_s3.pdf]

OXFORD
human reproduction
Human Reproduction, 2025, 1–1
https://doi.org/10.1093/humrep/deaf181
Supplementary Data

Supplementary Table S3. Left, right, and averaged uterine artery Doppler indices by gestational age and CL group (means from raw measurements).

		0 CL			>1 CL			1 CL		
		Right	Left	Average	Right	Left	Average	Right	Left	Average
7 weeks	UtA PI	2.47	1.94	2.23	2.33	2.41	2.36	2.73	2.74	2.76
	UtA RI	0.83	0.79	0.81	0.83	0.83	0.83	0.87	0.86	0.87
9 weeks	UtA PI	2.03	1.98	2.03	2.01	2.11	2.04	2.15	2.20	2.17
	UtA RI	0.78	0.76	0.77	0.78	0.79	0.79	0.79	0.80	0.79
11 weeks	UtA PI	1.61	1.60	1.61	1.85	1.79	1.83	1.86	1.84	1.86
	UtA RI	0.69	0.68	0.68	0.75	0.74	0.75	0.74	0.74	0.74
22 weeks	UtA PI	0.68	0.65	0.66	0.91	0.93	0.92	0.87	0.92	0.90
	UtA RI	0.46	0.44	0.45	0.54	0.55	0.55	0.53	0.54	0.54
32 weeks	UtA PI	0.73	0.65	0.68	0.77	0.80	0.78	0.76	0.80	0.78
	UtA RI	0.48	0.45	0.46	0.49	0.50	0.50	0.49	0.50	0.49

CL, corpus luteum; PI, pulsatility index; RI, resistance index.
