# Supplementary material for: Corpus luteum number and maternal circulatory adaptation from early pregnancy onwards: the Rotterdam Periconception Cohort (Predict Study)
Source: Hum Reprod. 2025 Sep 16;40(11):2078–87. doi: 10.1093/humrep/deaf181 (PMC12584914; doi:10.1093/humrep/deaf181)
Supplement: deaf181_Supplementary_Table_S4 [file deaf181_supplementary_table_s4.pdf]

Human Reproduction, 2025, 1–1
https://doi.org/10.1093/humrep/deaf181
Supplementary Data

Supplementary Table S4. Presence of notching in uterine artery.

		0 CL n = 32	>1 CL n = 194	1 CL n = 398	P-value
7 weeks	No	0 (0.0%)	1 (0.5%)	2 (0.5%)	0.318
	Yes	10 (31.2%)	38 (19.6%)	65 (16.6%)	
	Missing	22 (68.8%)	155 (79.9%)	350 (82.9%)	
9 weeks	No	5 (15.6%)	29 (14.9%)	42 (10.6%)	0.416
	Yes	23 (71.9%)	125 (64.4%)	270 (67.8%)	
	Missing	4 (12.5%)	40 (20.6%)	86 (21.6%)	
11 weeks	No	12 (37.5%)	45 (23.2%)	79 (19.8%)	0.214
	Yes	15 (46.9%)	116 (59.8%)	248 (62.3%)	
	Missing	5 (15.6%)	33 (17.0%)	71 (17.8%)	
13 weeks	No	8 (25.0%)	17 (8.8%)	26 (6.3%)	**<0.001**
	Yes	2 (6.2%)	31 (16.0%)	102 (25.6%)	
	Missing	22 (68.8%)	146 (75.3%)	287 (68.1%)	
22 weeks	No	28 (87.5%)	143 (73.7%)	287 (72.1%)	0.266
	Yes	0 (0.0%)	23 (11.9%)	51 (12.8%)	
	Missing	4 (12.5%)	28 (14.4%)	60 (15.1%)	
30 weeks	No	22 (68.8%)	144 (74.2%)	267 (67.1%)	0.123
	Yes	0 (0.0%)	13 (6.7%)	38 (9.5%)	
	Missing	10 (31.2%)	37 (19.1%)	93 (23.4%)	
Persisting notch	No	21 (65.6%)	117 (60.3%)	227 (56.9%)	0.561
	Yes	0 (0.0%)	6 (3.1%)	19 (4.8%)	
	Missing	11 (34.4%)	71 (36.6%)	152 (38.2%)	

CL, corpus luteum. Bold values indicate statistical significance ($P < 0.05$).
