# Supplementary material for: Corpus luteum number and maternal circulatory adaptation from early pregnancy onwards: the Rotterdam Periconception Cohort (Predict Study)
Source: Hum Reprod. 2025 Sep 16;40(11):2078–87. doi: 10.1093/humrep/deaf181 (PMC12584914; doi:10.1093/humrep/deaf181)
Supplement: deaf181_Supplementary_Table_S5 [file deaf181_supplementary_table_s5.pdf]

OXFORD
human reproduction

Human Reproduction, 2025, 1–1
https://doi.org/10.1093/humrep/deaf181
Supplementary Data

Supplementary Table S5. Effect estimates of linear mixed models for difference in blood pressure and for 0 CL and >1 CL, for ART pregnancies only.

		0 CL		>1 CL	
		**Beta (95% CI)**	**P-value**	**Beta (95% CI)**	**P-value**
All trimesters					
Systolic blood pressure	Model 1	1.53 (−1.29–4.35)	0.286	−0.73 (−2.61 to 1.14)	0.445
	Model 2	1.56 (−1.14 to 4.26)	0.256	0.21 (−1.64 to 2.07)	0.821
Diastolic blood pressure	Model 1	3.50 (1.52–5.47)	**0.001**	−0.63 (−1.95 to 0.68)	0.345
	Model 2	3.20 (1.30–5.11)	**0.001**	−0.18 (−1.49 to 1.13)	0.784
Mean arterial pressure	Model 1	2.93 (0.85–5.00)	**0.006**	−0.64 (−2.01 to 0.74)	0.363
	Model 2	2.66 (0.70–4.63)	**0.008**	−0.06 (−1.41 to 1.29)	0.930

1 CL as reference group. Model 1: adjusted for gestational age. Model 2: adjusted for gestational age, maternal age at conception, BMI, nulliparity, smoking in periconception period, and pre-existing hypertension.
CL, corpus luteum. Bold values indicate statistical significance ($P < 0.05$).
