# Supplementary material for: Corpus luteum number and maternal circulatory adaptation from early pregnancy onwards: the Rotterdam Periconception Cohort (Predict Study)
Source: Hum Reprod. 2025 Sep 16;40(11):2078–87. doi: 10.1093/humrep/deaf181 (PMC12584914; doi:10.1093/humrep/deaf181)
Supplement: deaf181_Supplementary_Table_S6 [file deaf181_supplementary_table_s6.pdf]

OXFORD

human reproduction

Human Reproduction, 2025, 1–1
https://doi.org/10.1093/humrep/deaf181
Supplementary Data

Supplementary Table S6. Mean predicted uterine artery pulsatility index and resistance index for corpus luteum groups when restricted to ART pregnancies only.

		0 CL			>1 CL			1 CL	
		**Mean**	**95% CI**	**P-value**	**Mean**	**95% CI**	**P-value**	**Mean**	**95% CI**
7 weeks	UtA PI	2.01	1.73–2.34	**0.011**	2.25	2.09–2.41	0.067	2.57	2.28–2.89
	UtA RI	0.79	0.74–0.86	0.116	0.82	0.79–0.85	0.182	0.86	0.81–0.91
9 weeks	UtA PI	1.76	1.59–1.94	**0.005**	1.98	1.89–2.08	0.228	2.09	1.94–2.26
	UtA RI	0.74	0.71–0.78	**0.031**	0.78	0.76–0.80	0.450	0.79	0.77–0.83
11 weeks	UtA PI	1.51	1.37–1.67	**0.033**	1.74	1.66–1.82	0.786	1.72	1.61–1.84
	UtA RI	0.69	0.66–0.72	**0.025**	0.74	0.73–0.76	0.633	0.74	0.71–0.76
22 weeks	UtA PI	0.63	0.56–0.71	**<0.001**	0.88	0.84–0.92	**0.045**	0.80	0.74–0.86
	UtA RI	0.44	0.41–0.46	**<0.001**	0.54	0.53–0.55	**0.008**	0.51	0.49–0.53
32 weeks	UtA PI	0.68	0.60–0.77	0.917	0.72	0.68–0.76	0.340	0.69	0.64–0.74
	UtA RI	0.46	0.44–0.50	0.951	0.48	0.46–0.49	0.235	0.46	0.45–0.48

Based on adjusted linear mixed models, side = right, no smoking, no pre-existing hypertension, mean BMI and mean age. 1 CL as reference group. Adjusted for maternal age at conception, BMI, nulliparity, smoking in periconception period, and pre-existing hypertension.
CL, corpus luteum; PI, pulsatility index; RI, resistance index. Bold values indicate statistical significance ($P < 0.05$).

© The Author(s) 2025. Published by Oxford University Press on behalf of European Society of Human Reproduction and Embryology.
This is an Open Access article distributed under the terms of the Creative Commons Attribution License (https://creativecommons.org/licenses/by/4.0/), which permits unrestricted reuse, distribution, and reproduction in any medium, provided the original work is properly cited.
Human Reproduction, 2025, 00, 1–16
https://doi.org/10.1093/humrep/deaf181
Original Article
